# Impact of Strength Parameters and Material Structure of Bone Plates on Displacement of Bone Fragments in the Injured Area

**DOI:** 10.3390/jfb16020044

**Published:** 2025-01-29

**Authors:** Arkadiusz Szarek, Grzegorz Golański, Zbigniew Bałaga, Marcin Godzierz, Mariusz Radecki

**Affiliations:** 1 Department of Technology and Automation, Faculty of Mechanical Engineering and Computer Science, Czestochowa University of Technology, 21 Armii Krajowej Av., 42-201 Czestochowa, Poland; 2 Faculty of Production Engineering and Materials Technology, Czestochowa University of Technology, 19 Armii Krajowej Av., 42-201 Czestochowa, Poland; grzegorz.golanski@pcz.pl (G.G.); zbigniew.balaga@pcz.pl (Z.B.); 3 Centre of Polymer and Carbon Materials, Polish Academy of Science, M. Curie-Skłodowskiej Street 34, 41-819 Zabrze, Poland; mgodzierz@cmpw-pan.pl; 4 Orthopedics and Traumatic Surgery Department of NMP Voivodship Specialist Hospital in Czestochowa, Bialska Street 104/118, 42-200 Czestochowa, Poland; mariuszradecki@szpitalparkitka.com.pl

**Keywords:** compression plate, microstructure, aging tests, biomaterials strength, autocompression plate, dynamic compression plate

## Abstract

The study is a metallographic analysis of commercial bone plates used for stabilizing long bones. The plates examined were delivered to the hospital in different years, and the course of treatment of patients with similar goniometric and anthropometric parameters varied dramatically. To determine the characteristics of displacement of bony fragments in the area of the simulated fracture and relate it to the strength parameters of the bone plate, experimental tests were carried out on composite femurs loaded according to the biomechanical loading model at known values of forces acting on the femoral head. In order to assess the influence of material parameters of the plate on the biomechanics of the bone–bone plate system, microstructural and strength tests were performed, i.e., three-point bending tests, chemical composition and hardness assessments, as well as evaluation of the state of internal stresses in the tested materials. The research conducted allowed us to develop guidelines for companies producing bone fixations and orthopedic surgeons who use bone plates to stabilize bones after mechanical trauma, allowing the plates to be tailored to individual patient characteristics.

## 1. Introduction

The concept of internal fixating structures dates back to the mid-19th century, but it became widely popular in the 1960s [1] and has remained largely unchanged since then. Plate fixations are used for multiple areas of bone virtually throughout the entire bony skeleton and are designed to restore bone shape congruence, provide stable immobilization of the fragments and achieve the best possible adhesion to maximize patient performance and avoid complications [2]. The main purpose of such a solution is dynamic stabilization, the premise of which is flexibility of the structure to accelerate the healing process. The fixation of bone fragments involves restoring the shape of the bone and is not a major problem, but from a biomechanical point of view, a serious problem arises when the bone carries significant mechanical loads resulting, for example, from motor activity.

Dynamic compression plating is a fundamental type of bone fracture fixation used for generating interfragmentary compression [3].

Currently, the main direction of research is carried out to determine the causes of plate damage during the service life [1,4,5] or numerical simulation of the stress and strain condition of the bone-plate system under a load [6,7,8,9].

Periosteal osteosynthesis promoted by ASIF (AO) [10] has certain advantages as well as numerous disadvantages [11]. Its advantages include a relatively easy surgical technique and a wide range of application of this method, but the stiff method of stabilization is the reason for adhesion complications that are difficult to treat. Bone inflammation, proliferation of infected pseudo-avascular joints and demineralization of bone or loss of its load-bearing properties may occur. Extensive intraoperative debridement of the periosteum promotes adhesion disorders. This method also causes ischemia of bone fragments, which may be the result of a massive structure of the fastener or faulty surgical technique. The unfavorable biological and biochemical properties of the rigid fixation may also be a disadvantage. It is one of the prerequisites for primary bone adhesion and is only possible after the bone fragments have been accurately set and adequate pressure has been achieved among the bone fragments. Bone atrophy, in turn, resulting from extraction of the plate, can cause a repeat fracture in a given area. There is also the possibility of cracking the plate in the area of maximum material strain, recommended by the AO, stimulating the development of corrosion. The disadvantages mentioned above are unavoidable unless the principle of fixation, that is, direct attachment of the plate to the bone, is changed [12,13]. As one can see, the correct selection of the strength parameters of the bone plates is important not only for maintaining the proper stiffness of the system but also because it has a decisive impact on the course of treatment and on the potential complications.

Accordingly, the main research problem of the study was outlined, namely that the course of bone adhesion after a long bone fracture demonstrates that patients with similar anthropometric and goniometric parameters as well as age have significant differences in bone superstructure and the period of bone adhesion. An attempt was made to determine whether the stiffness of the bone-stabilizer system and the strength parameters of the stabilizer plate can significantly affect the adhesion process.

The main research thesis of the study is that the decisive influence on the displacement of bone fragments in the fracture area and, consequently, on the course of adhesion, is due to the different strength parameters of the bone plates made of the same biomaterials.

The clinical material selection occurred over three years. Bone plates were supplied by one manufacturer but from different production periods. Such extensive research enabled knowledge acquisition about the displacement of bone fragments in plates used for comparable periods (up to 16 weeks) by individuals with similar anthropometric and goniometric parameters. The number of plates selected for the study was limited to ensure the comparability of operational parameters. During the entire treatment process, the operational area was aseptic, meaning there were no infections, inflammations, or any combination of biological, chemical, or mechanical factors resulting from the body’s reaction to the fixation. Based on the elements removed from the human body, displacement of the fragments in the area of injury at known loads on the femoral head was simulated, and the basic material and structural parameters affecting the course of micro-movements of the bone fragments were analyzed.

Manufacturers conduct basic mechanical tests such as tensile, compression, and impact strength to meet material standards, so such data already exist. The work has shown that the same product delivered to the hospital by the same company but in different production periods may have different operating parameters. This is in accordance with the law and ISO/EN material standards (because material standards provide a certain range of use of individual chemical elements, and the degree of strengthening, such as after plastic processing of the material, is not indicated on the product). Such information is essential from the point of view of the displacement of bone fragments during limb loading. For example, according to the standard, austenitic stainless steels have strength at the level of tensile strength, from 450 to 1850 MPa. All materials in this range meet the requirements, but if such information—suitably unified, e.g., on a scale from 1 to 10—was available to the end user, it would be possible to better select a plate for the patient. Supplementing the research with the tests presented in this work does not entail significant costs while providing considerable information about the product’s operating conditions, crucial for the fracture healing and treatment process.

The Brinell hardness test is affordable and universal, providing information about the material’s structure and hardness. Gathering such data allows for long-term monitoring of production consistency and even integrating the data into a simple artificial intelligence module overseeing and optimizing quality control. This testing is cost-effective and quick, offering fundamental knowledge about the material microstructure (semi-finished and final products).

## 2. Research Material and Methods

In order to evaluate the strength differences in bone plates affecting the process of adhesion of damaged bone, tests were conducted on plates removed from the human body. The study was carried out in cooperation with the Department of Orthopedics and Traumatology of the Locomotor System of the Holy Virgin Mary Province Specialized Hospital in Czestochowa. The bone plates removed after a comprehensive period of treatment, in different periods of operation, were transferred to the Czestochowa University of Technology, where simulation, strength and microstructural tests were carried out.

A certified bone composite, specifically a femur, from the Swedish company Sawbones was used for the study. It was a fourth-generation composite medium left, to which dedicated bone bars were screwed in. Bone screws were tightened with a force of 15 Nm, which corresponded to a lateral thrust of 750 N, according to AO guidelines [14]. Since plates with 8 fixation screws were obtained from the analyzed clinical material, it can be assumed that the value of the fixation force is 8 × 750 = 6000 N, distributed over an area of about 20% of the plate, which should maintain a stable connection of the bone-stabilizer system.

### 2.1. Simulation of Bone Fragment Displacement

**Figure 1:**
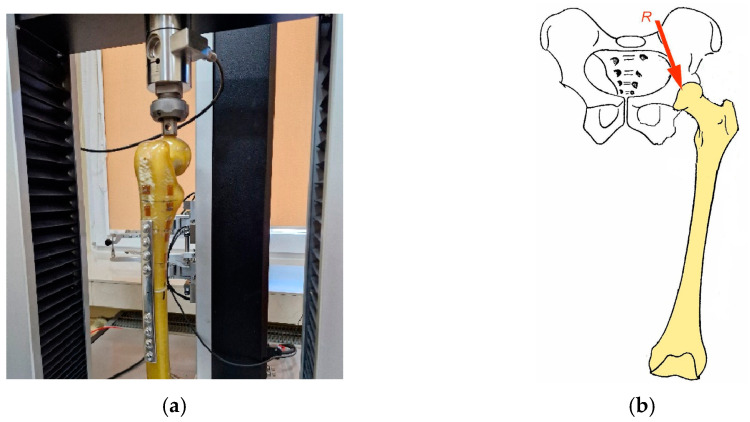
Experimental model of the bone-stabilizer system, (**a**) force application, (**b**) loading model.

To determine the displacement of bone fragments within the simulated injury in the experimental model, a crack was developed to simulate a bone fracture. A symmetrical crack was modeled in the horizontal plane across the bone separating the splinters at a distance of 5 mm. The plates selected for the study measured 170 [mm] in length (L), 15 [mm] in width (B), and 4 [mm] in thickness (s), product manufacturer: MEDGAL Sp. z o.o. KSIEZYNO, POLAND. The load application site and restraints were adopted according to the model of static thigh loading resulting from standing on both feet. The main assumptions considered in loading the femoral head (Figure 1a) were mapped according to the Pauwels model (Figure 1b), where R wasthe resultant force acting on the femoral head [15,16].

**Figure 2:**
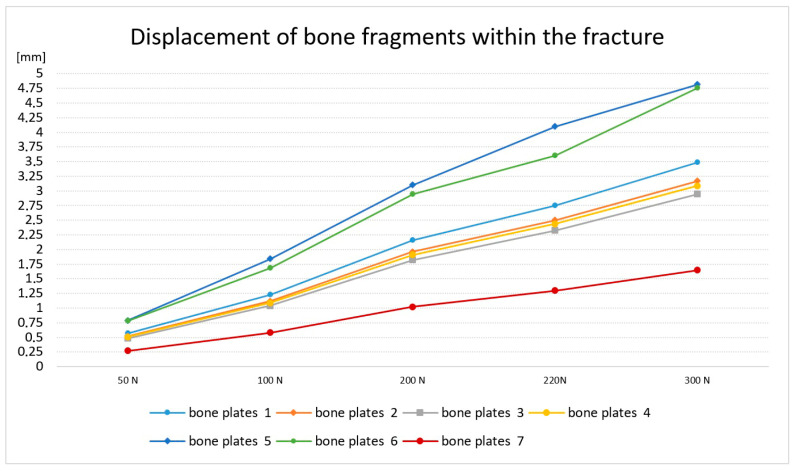
Displacement of bone fragments within the fracture.

The bone was restrained within the distal metaphysis, and a static force was applied to the femoral head. Bone displacement within the crack was determined and calculated according to the rules of experimental planning and statistics for 10 repetitions for each of the bone plates tested. The following loads were assumed for the study: R_1_ = 50 [N], R_2_ = 100 [N], R_3_ = 200 [N], R_4_ = 220 [N] and R_5_ = 300 [N]. The displacement of bone fragments relative to each other was analyzed. The load R_4_ = 220 [N] was determined experimentally, since at this load, more than half of the specimens experienced a halving of the simulated crack. The course of plate deformation and the change in the width of the crack are shown in Figure 2.

Based on the study, it was found that the course of the bone crack closure for most of the bone plates was similar, but one sample had a significantly lower strain, and two plates had a disproportionately high strain, and thus very high crack closure. It was also found that with a load of 220 N, for most of the analyzed plates, there is a significant reduction in the crack. Therefore, in the respective plates, the displacement is, respectively,

P_r1_ = 2.75 mm; (2), P_r2_ = 2.5 mm; P_r3_ = 2.33 mm, P_r4_ = 2.44 mm (1), P_r5_ = 4.1 mm, P_r6_ = 3.6 mm (3) and P_r7_ = 1.3 mm.

According to the rules of experimental planning and statistics, the samples with extreme values of deformation were rejected. Two specimens with similar strain values and one specimen with the highest strain were selected for further testing.

In order to determine the cause of such large differences in the displacement of bone fragments, metallographic tests were carried out for the selected plates. Preliminary studies have shown that bone plates with similar strength parameters are characterized by an analogous structure, and further studies have been adopted, i.e., plate numbers 1, 4 and 6.

### 2.2. Metallographic Research Methodology

The chemical composition of the tested elements was carried out using a Bruker Q4 Tasman spark spectrometer (Bruker, Billerica, MA, USA). Microscopic examinations were carried out using a Keyence VHX 7000 digital microscope (Keyence, Osaka, Japan) on metallographic specimens etched with aqua regia. The hardness of the test samples was measured using the Brinell method with a carbide ball with a diameter of 2.5 mm using a load of 187.5 kG. The Brinell hardness was measured using a KB 250 hardness tester (KB Prüftechnik GMBH, Hochdorf-Assenheim, Germany). The three-point bending test was carried out using a Zwick/Roell Z100 universal testing machine (Zwick Roell, Ulm, Germany). The analysis of self-stress in the test plates was carried out using a Bruker D8 Advance X-ray diffractometer (Bruker, Billerica, MA, USA). An X-ray tube with a copper anode was used for the test. The measurements were carried out in accordance with the guidelines provided in EN-15305 [17]. The plates selected for the study measured 170 mm in length (L), 15 mm in width (B) and 4 mm in thickness (s). The number of plates selected for the study was limited to ensure the comparability of operational parameters. The plates under examination were used in the body for no longer than 16 weeks.

## 3. Research Results and Their Discussion

An analysis of the chemical composition of the analyzed pieces showed that they were made of austenitic stainless steel in grade X2CrNiMo18-14-3 with the composition shown in Table 1, Table 2 and Table 3.

Steel of this grade is classified as a corrosion-resistant material. In addition to the addition of chromium and nickel, the chemical composition of these alloys included additions of molybdenum and nitrogen at levels indispensable for the formation of an austenitic structure and for obtaining the required corrosion resistance. Molybdenum and nitrogen content in the chemical composition of austenitic stainless steels increase the resistance of this group of materials to local corrosion, specifically pitting corrosion. The pitting corrosion resistance of austenitic stainless steels can be determined by the equivalent Pitting Resistance Equivalent Number (PREN), the value of which depends on the chemical composition of the material, i.e., the content of chromium, molybdenum and nitrogen (Equation (1)) [18].

(1) PREN=%Cr+3.3%Mo+16%N.

The calculated PREN equivalent value for the alloys tested was 27.65 ÷ 31.3, higher than the value of 26 required for these alloys when used as biomaterials.

**Figure 3:**
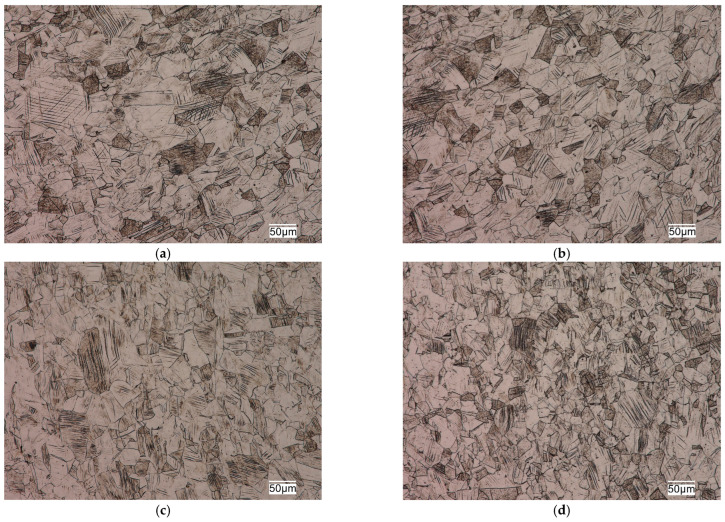

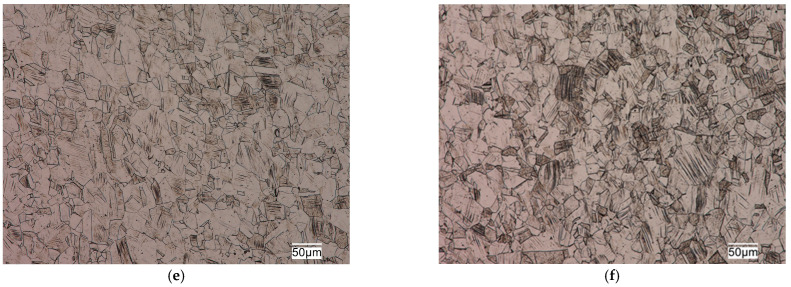
Examples of alloy microstructures: (**a**,**b**) No. 1; (**c**,**d**) No. 2; (**e**,**f**) No. 3. Left row—in the fixation area; right row—in the central part of the plate.

The tested sections were characterized by a fine-grained austenitic structure with numerous twins (Figure 3). No delta ferrite precipitates were observed in the structure of the analyzed materials. The microscopic observations made, indicating the single-phase structure of the analyzed materials, were confirmed by calculations of the values of nickel equivalent Ni_e_ (2) and chromium equivalent Cr_e_ (3). Chromium and nickel equivalents determine the effect of dissolved elements in the matrix on the microstructure of corrosion-resistant steels [18].

(2) $${\mathrm{Ni}}_{e}=\%\mathrm{Ni}+\%\mathrm{Co}+0.5\%\mathrm{Mn}+0.3\%\mathrm{Cu}+30\%C+25\%N,$$

(3) $${\mathrm{Cr}}_{e}=\%\mathrm{Cr}+2\%\mathrm{Si}+1.5\%\mathrm{Mo}+5\%V+5.5\%\mathrm{Al}+1.75\%\mathrm{Nb}+0.75\%W.$$

The calculated grain size in the analyzed steels was as follows: steel No. 1–6, steel No. 2–7.5 and steel No. 3–6, according to the scale of ASTM standards (included in EN ISO 643 [19]). The grain size of austenitic stainless steels does not have as significant an effect on their mechanical properties as it does for ferritic matrix steels. However, the fine grain size in this group of alloys not only provides higher plastic properties, ductility and fatigue resistance, but also reduces the susceptibility of these alloys to brittle fracture and has a positive effect on increasing their corrosion resistance.

Austenitic stainless steels, due to their mesh type and low misalignment energy, are characterized in the supersaturated state (delivery state) by relatively low strength properties with very high plastic and ductility properties. This is mainly due to the strengthening mechanism that is fundamental to this group of materials in the delivery state, i.e., the solid solution mechanism of strengthening. The degree of strengthening is related to the concentration of the main alloy additives, but the most effective strengthening comes from the interstitial elements, namely nitrogen and carbon. Based on the mechanism of solid solution strengthening, it is possible to calculate for austenitic stainless steels the approximate value of yield strength (R_p0.2_) using relationship (4) [20].

(4) $$R_{p0.2}=77\sqrt{N}+20\mathrm{Mn}+7\mathrm{Cr}+33\mathrm{Si}+2.9\mathrm{Ni}+(0.24+1.1N)\frac{1}{\sqrt{d}}$$

where d is the average grain diameter, measured in mm.

For the alloys studied, the calculated value of the yield strength in the supersaturated state, due to their similar chemical composition (Table 1, Table 2 and Table 3), was comparable, with a value of about 240 MPa for the analyzed alloys. The calculated value of this parameter was higher than the normative requirements for this grade of steel (Table 4).

In practice, the wide range of values of strength parameters (permissible in the normative range [15]) (Table 4) of this steel grade makes it possible to market plate stabilizers that are diametrically different in terms of operation within the body. In a bone-plate system, with the strength parameters of the stabilizer material being standard-compliant but extreme, the possibility of deformation will be dramatically different.

Slip lines of varying intensity were also observed in the respective grains in the microstructure of these alloys (Figure 3). The presence of numerous slip lines in individual austenite grains indicates cold plastic deformation of these sections. Plastic deformation of austenitic stainless steels is one of the cheap and simple mechanisms to strengthen them through an increase in dislocation density. The increase in the density of dislocations in the metal matrix contributes to limiting the mobility or blocking the dislocations entirely. It causes an increase in the steady stress that is necessary for making these defects move, which results in the strengthening of the material. Strengthening of this type results in a significant increase in strength properties with a decrease in plasticity properties and ductility, and depends on the degree of deformation of the alloy. The structural effect of strain hardening is the presence of numerous slip lines and bands in the microstructure of austenitic stainless steels, which was evident in the microstructure of the alloys studied, as seen in Figure 3.

The material strengthened by crushing will have a different level of mechanical properties than the material in the delivery state (after supersaturated heat treatment). The selected mechanical properties and stress condition of the tested materials are shown in Table 5. The data summarized in Table 5 indicate that the small displacement of the bone plate during the experiment was probably related to the strengthening condition of a given material, namely austenitic stainless steel. The strengthening condition was related to the presence in the analyzed materials of stresses from the technological process of manufacturing these components. The compressive stresses shown in plates No. 1 and 2 translated into higher strength properties and hardness. The results of Brinell hardness measurements (Table 5) also confirm the higher degree of strengthening of alloy No. 1 in comparison with the remaining analyzed plates No. 2 and No. 3. Higher strength properties (and, also, Brinell hardness), in turn, resulted in lower deformation capacity of these plates, resulting in less displacement (Table 5).

## 4. Summary and Conclusions

On the basis of the tests conducted, it was discovered that all the specimens used in the tests were made of austenitic stainless steel complying with PN EN 10088-2 [21], but with different degrees of strengthening, most likely due to the manufacturing process of the analyzed elements.

The samples obtained from patients after a comprehensive treatment process at different delivery times are characterized by different strength parameters that have a decisive impact on the biomechanics of bone fragments during the loading of the limb in patients with similar anthropometric and goniometric parameters.

This may indicate that the strength parameters of the bone plates may vary depending on the batch of the delivered semi-finished products or that the manufacturer obtains different strength parameters of the product as a result of technological processes.

The study demonstrates that, with relatively low cost and fairly simple strength testing, it is possible to personalize the bone plates to match their stiffness and strength to a selected group of patients. In order to make it easier to customize plates for individual fixations, it is suggested to choose not only different plate sizes, shapes, or number of fixation screws, but also to introduce new additional markings, allowing to adjust plate stiffness, for example, to the patient’s Body Weight. Determining the displacement characteristics of bone fragments and the deformation of the bone–bone plate system does not require complex or expensive tests from manufacturers. The proposed set of tests in the developed load range is fully comprehensive and will make it possible to determine for each batch of devices what deformations of the bone–bone plate system will be generated when the patient moves. Proper labeling and communication with the orthopedic surgeon will allow for selecting the stiffness of the fixation so that it stimulates bone adhesion rather than rupturing it or, in extreme cases, producing an overly stiffened system that would contribute to complications that are difficult to treat.

Recommendations for companies that produce bone plates and for physicians are as follows.

- Each batch of plates marketed should be tested not only for material and strength testing, but also for simulated wear testing with control of bone fragment behavior.
- Markings should be introduced in production to allow for the personalization of the device to individual patient needs, e.g., by experimentally determining the displacement of bone fragments for the specific body weight (BW) of the patient.
- Each batch of the products should be marked with unambiguous strength parameters relating to, for example, the patient’s BW, so that the orthopedic surgeon can tailor the plate as precisely as possible to specific patient characteristics.
- For patients with non-standard BW or complex osteoarticular pathologies, personalized stabilization plates with bone surface shape and strength parameters tailored to the individual patient’s needs are recommended.
- Guidelines for physicians are as follows.
- Select bone plates adapted to the individual goniometric and anthropometric characteristics of the patient.
- Adjust the bone plates not in terms of external dimensions but in terms of strength determined experimentally under controlled restraint and load parameters.
- Obtain information from manufacturers on the biomechanics of bone fragments in the fracture area with a simulated, fully controlled stress test of the bone–bone plate system.

## Figures and Tables

**Table 1:** Chemical composition of alloy No. 1, %mass.

C	Si	Mn	P	S	Cr	Mo	Ni	N
0.024	0.21	1.95	0.017	0.005	17.00	2.59	15.51	0.11

**Table 2:** Chemical composition of alloy No. 2, %mass.

C	Si	Mn	P	S	Cr	Mo	Ni	N
0.019	0.21	1.97	0.021	0.005	16.63	2.58	15.69	0.10

**Table 3:** Chemical composition of alloy No. 3, %mass.

C	Si	Mn	P	S	Cr	Mo	Ni	N
0.014	0.20	1.95	0.018	0.004	17.05	2.58	15.51	0.11

**Table 4:** Required mechanical properties of X2CrNiMo18-14-3 steel in delivery condition [21].

YS MPa	TS MPa	El. %	KV J	E GPa
min. 220	550 ÷ 700	min. 40	min. 90	200

YS—yield strength; TS—tensile strength; El.—elongation; KV—impact energy; E—Young’s modulus.

**Table 5:** Summary of the results of tests of mechanical properties and stress condition.

Designation Samples	Displacement P_r_ mm	Maximum Force in Bending F_max_ N	dL at F_max_ mm	HBW 2.5/187.5	Stresses δ_11_ MPa
Plate 1 (pr2)	2.44	7250	9.80	293	−311
Plate 4 (pr1)	2.76	8560	9.80	282	−136
Plate 6 (pr3)	3.60	4410	17.40	271	52

HBW 2.5/187.5—Brinell hardness, H from hardness, B from Brinell, and W from the material of the indenter, tungsten (wolfram) carbide; 2.5 mm—diameter of indenter; 187.5 kG—applied load in kilogram-force.

## Data Availability

The original contributions presented in the study are included in the article, further inquiries can be directed to the corresponding author.
